# miRNAs and Other Epigenetic Changes as Biomarkers in Triple Negative Breast Cancer

**DOI:** 10.3390/ijms161226090

**Published:** 2015-11-30

**Authors:** Andrea Mathe, Rodney J. Scott, Kelly A. Avery-Kiejda

**Affiliations:** 1 Centre for Information Based Medicine, Hunter Medical Research Institute, Newcastle, NSW 2305, Australia; andrea.mathe@uon.edu.au (A.M.); rodney.scott@newcastle.edu.au (R.J.S.); 2 Priority Research Centre for Cancer, School of Biomedical Sciences and Pharmacy, Faculty of Health, University of Newcastle, Newcastle, NSW 2308, Australia; 3 Hunter Area Pathology Service, Pathology North, John Hunter Hospital, Newcastle, NSW 2305, Australia

**Keywords:** microRNA, triple negative breast cancer, DNA methylation

## Abstract

Triple negative breast cancer (TNBC) is characterised by the lack of receptors for estrogen (ER), progesterone (PR), and human epidermal growth factor 2 (HER2). Since it cannot be treated by current endocrine therapies which target these receptors and due to its aggressive nature, it has one of the worst prognoses of all breast cancer subtypes. The only treatments remain chemo- and/or radio-therapy and surgery and because of this, novel biomarkers or treatment targets are urgently required to improve disease outcomes. MicroRNAs represent an attractive candidate for targeted therapies against TNBC, due to their natural ability to act as antisense interactors and regulators of entire gene sets involved in malignancy and their superiority over mRNA profiling to accurately classify disease. Here we review the current knowledge regarding miRNAs as biomarkers in TNBC and their potential use as therapeutic targets in this disease. Further, we review other epigenetic changes and interactions of these changes with microRNAs in this breast cancer subtype, which may lead to the discovery of new treatment targets for TNBC.

## 1. Introduction

Breast cancer has the highest incidence rate of all cancers in women worldwide [1]. It is a very heterogeneous disease and there are multiple ways by which to classify breast cancer into its subtypes. However, the primary diagnosis remains the histopathology report of the tumour which assesses the presence or absence of hormone receptors for estrogen (ER), progesterone (PR), and the human epidermal growth factor receptor-2 (HER2). The expression of these receptors is required to determine the patients’ suitability for endocrine therapies such as Tamoxifen, Anastrozole, and Trastuzumab [2]. The majority of breast cancers are receptor positive (77% [3]) and targeted treatment has proven efficacy. However, in the case of breast cancers that are negative for all three receptors (triple negative breast cancers, TNBC) there is, as yet, no targeted treatment available [4].

Further characteristics of the TNBC subtype are: germline *BRCA1* mutations (10%) [5], high mitotic counts and TP53 positivity [6]. The majority of TNBCs are from the basal-like subtype (~70%) [7] and express basal-type cytokeratin 5 and cytokeratin 6, as well as high expression of the epidermal growth factor receptor (*EGFR*) [8]. Most TNBCs are classed as invasive ductal carcinomas, nevertheless a high proportion of other histology types are ER, PR, and HER2 negative including metaplastic carcinomas [9,10] and apocrine carcinomas [11]. TNBC accounts for only 10%–17% of all breast cancer patients [12]. However, the disease is more common in young women (under 40 years of age/pre-menopausal [13]) and especially in African-Americans [12]. TNBC has a poor prognosis, associated with an increased number and earlier appearance of metastases (on average within the first 2.6 years after diagnosis [14]) compared to other breast cancer subtypes [6]. Within this review, we aim to provide an overview of the current knowledge regarding miRNAs and other epigenetic mechanisms that are involved in the development and progression of TNBC.

### Clinical Trials in TNBC

The lack of hormone receptors and HER2 significantly reduces targeted treatment options for patients with TNBC. At the moment, the only available treatments are chemotherapy and surgery [12]. There are some trials with poly (ADP-ribose) polymerase (PARP) inhibitors, angiogenesis inhibitors, EGFR-targeted agents, src kinase inhibitors, and androgen receptor inhibitors [6,15], but none display significant improvements in all TNBC cases pointing to the heterogeneity of disease. PARP-inhibitors have shown the most encouraging results in that there are good responses in TNBC-patients who harbour a *BRCA1* mutation, but not in others. This can be explained by the requirement for both genes (*BRCA1* and *PARP*) to be engaged in DNA repair, so if both fail to function any DNA damage will not be repaired and the cell undergoes apoptosis [16]. A phase 2 clinical trial with the PARP-inhibitor iniparib showed promising results, increasing clinical benefit of chemotherapy from 34% to 56% and the rate of overall response to chemotherapy from 32% to 52%, overall survival was improved from 7.7 to 12.3 months [17]. Unfortunately the subsequent phase 3 clinical trial was negative and did not meet the criteria for progression-free survival and overall survival [18]. Angiogenesis inhibitors are being tested in TNBC patients since they show a high level of intra-tumoral vascular endothelial growth factor (VEGF) compared to non-TNBC patients [19]. A meta-analysis of three clinical trials (E2100, AVADO, RIBBON-1), testing a VEGF inhibitor (bevacizumab) in combination with chemotherapy, revealed improved progression-free survival (8.1 months *versus* 5.4 months), a change in relative risk (42% *versus* 23%), but no overall survival benefit [20]. About 45%–70% of TNBC patients show epidermal growth factor receptor (*EGFR*) over-expression [21]; which has led to clinical trials of EGFR-targeted therapies. The combination of cetuximab (an EGFR inhibitor) and chemotherapy increased the overall response rate from 10% to 20%, progression-free survival from 1.5 to 3.7 months and overall survival from 9.4 to 12.9 months [22]. Src tyrosine kinase inhibitors have been tested, as the Src tyrosine kinase is often over-expressed in aggressive breast cancers [23]. However, Src inhibitors like dasatinib have almost exclusively been tested in cell lines and not in patients [24]. A phase 1 clinical trial showed a partial response in 31% of patients and 29% of patients had stable disease after treatment [25]. The androgen receptor is expressed in most breast cancers, including TNBC (70%) [26] and its role in TNBC has recently been reviewed [27]. Clinical trials for androgen receptor inhibitors for the targeted treatment of TNBC are still in early stage development. In a phase 2 study, in patients with ER/PR-negative and androgen receptor positive advanced breast cancer, the anti-androgen agent bicalutaminde has shown a 6 month clinical benefit rate of 19% and the median progression-free survival was 12 weeks [28].

There has been growing interest in DNA damaging agents such as platinum agents (cisplatin and carboplatin) as a treatment option for TNBC. As described above, TNBC is associated with *BRCA* mutations and a high proliferation rate which increases the sensitivity of this subtype to interstrand cross-linking agents such as platinum agents, when compared to other breast cancer subtypes. A recent systematic review by Petrelli *et al.* [29] on 28 studies that investigated platinum based neoadjuvant therapy in TNBC patients showed that TNBC patients receiving platinum based neoadjuvant therapy have a significantly increased pathological complete response rate, compared to those not receiving neoadjuvant therapy. Further, they were able to show that TNBC patients have a threefold increased pathological complete response rate compared to non-TNBC subtypes.

## 2. Better Classification of TNBC to Find New Treatment Targets and Prognostic Indicators

Molecular subtypes of breast cancer have been based on gene expression patterns. Breast cancers were first divided into 4 subtypes: ER+/luminal-like, basal-like, HER2-enriched and normal breast-like [30] in 2000. However, this has been further defined in 2007 as six different subtypes with luminal-like now further subdivided into luminal A and B, and the additional classification of a claudin low subtype [31]. A report by Prat and Perou in 2011 showed that the majority of TNBCs are from the basal-like subtype (49%), followed by the claudin-low subtype (30%), then the HER2-enriched (9%), luminal A (5%), luminal B (6%), and normal-like (1%) subtypes [32]. However, with the advent of new technologies such as massively parallel sequencing it is likely that further subtypes will be revealed [33].

The heterogeneity of and difficulty in treating TNBC led Lehmann *et al.* to define six TNBC subtypes to provide the necessary discrimination for the development of new molecular-based treatment options [34]. The six subtypes, based on gene expression analysis, include two basal-like (BL1 and BL2), an immune-modulatory, a mesenchymal, a mesenchymal stem-like, and a luminal androgen receptor subtype. However, there were still samples that could not be categorized into one of these six subtypes (12%). Thus, a better classification scheme of these tumours is still required to accurately classify women with TNBC. More recently, a study by Burstein *et al.* [35] decreased the number of TNBC subtypes to four (luminal androgen receptor, mesenchymal, basal-like immunosuppressed, and basal-like immune-activated). They analysed RNA and DNA profiles from 198 TNBC samples and confirmed their results in seven external publically available datasets. This study also identified subtype specific targets, for the luminal androgen receptor subtype: the androgen receptor, and the cell surface mucin MUC1, for the mesenchymal subtype: growth factor receptors PDGFRA and c-Kit, for the basal-like immunosuppressed subtype: an immunosuppressing molecule VTCN1 and lastly for the basal-like immune-activated subtype: STAT signal transduction molecules and cytokines.

The heterogeneity in gene expression profiles is associated with distinct outcomes in TNBC patients. A recent study by Prat *et al.* used 1055 TNBC samples from all intrinsic TNBC subtypes. Prat *et al.* [36] revealed that TNBC patients with high expression of the basal-like or low expression of the luminal A signature were associated with a pathological complete response and improved survival following chemotherapy. Another study performed global proteomic profiling on two independent TNBC cohorts and identified one protein associated with good prognosis (trpRS) while two proteins were poor prognostic markers (DP, TSP1) [37]. Another study by Shen *et al.* (2014) shows the difficulties in identifying prognostic markers for TNBC. They tested for lymph node status, age, tumour size, histological grade, lymphovascular invasion, P53 status, Ki-67 index, and type of surgery, and found that only lymph node status was marginally significantly associated with poor prognosis. They also identified three miRNAs that were significantly higher expressed in metastatic TNBC compared to the disease-free group [38]. A further issue is that most studies only identify differences between tumours without identifying initial changes from the healthy/normal tissue that lead to cancerous growth and tumour initiation. This is partly due to the fact that the availability of healthy tissue is limited in research. Another approach is the use of normal adjacent tissue, which has been shown to have an expression profile characteristic of DNA repair deficiency [39,40]. Altogether, this evidence unequivocally indicates that a better classification scheme that can be reliably used to subdivide all TNBC samples into their respective groups depending on their prognosis and that is distinct from normal breast tissue is required to provide the appropriate information to tailor treatment options.

There has been an enormous amount of new research in recent years focusing on genetic and epigenetic changes associated with the development and progression of breast cancer. In this review we will focus on some of these changes especially microRNAs, DNA methylation and gene expression changes in TNBC.

## 3. Epigenetic Changes in TNBC—New Biomarkers?

One approach to identify biomarkers for TNBC is the analysis of epigenetic changes. Epigenetics is the study of heritable changes in the phenotype that does not involve any change in DNA sequence. In 1942 Conrad H. Waddington coined the words “epigenesis” and “genetics” to epigenetics to describe the “causal mechanisms” by which “the genes of the genotype bring about phenotypic effects” [41]. Due to the lack of experimental tools and overall knowledge it took over 50 years until scientists started to understand the underlying mechanisms of Waddington’s observations [42]. To date, multiple discoveries have been made revealing epigenetics can change a phenotype without altering the DNA sequence. These are the classical epigenetic mechanisms like histone modification, chromatin remodelling, and DNA methylation and also the more recently discovered epigenetic changes through small/non-coding RNAs such as miRNAs. These have been extensively reviewed in [43].

There remain many open questions about the mechanisms involved in epigenetic control but it is recognised that epigenetic change can occur due to environmental factors such as stress and cell damage. But there is little known about how genes are activated only when they are required. An increase in the understanding of epigenetic mechanisms and their contribution to disease development has led to a growing interest in the field of epigenetics. For the remainder of this review we will focus on microRNAs and DNA methylation control specifically associated with triple negative breast cancer.

## 4. MicroRNAs

MicroRNAs (miRNAs) are small (18–21 nucleotides) non-coding RNAs, which are capable of altering gene expression post-transcriptionally. In 1993, the lin-4 miRNA was discovered in *Caenorhabditis elegans*, where it was shown to decrease the levels of Lin-4 protein, by binding to the 3′ UTR region of its respective mRNA sequence [44]. Since that ground-breaking finding, microRNAs have been found to be highly conserved between species, suggesting they play a universal role in the regulation of gene expression. miRNAs regulate multiple biological processes including proliferation, cell death, development, and genomic stability [45]—all essential for tumour development. They not only regulate physiological conditions but also pathological ones, such as those involved in malignancy [46,47]. More than 2500 mature miRNAs have been identified in humans (miRBase v21) [48], but the functionality of most is yet to be discovered. One miRNA can interact with multiple (>100) target genes and one gene can be controlled by multiple miRNAs [49]. More than 60% of all protein coding genes have conserved miRNA binding sites in their 3′ UTR region, which affords them the possibility of control by their respective miRNAs [50].

miRNAs offer several advantages for expression analysis compared to mRNA: (1) they are small and therefore more stable; (2) they can be extracted from frozen tissue, formalin-fixed paraffin-embedded tissues as well as blood, with little/no degradation, which makes them ideal for clinical purposes, especially in relation to TNBC diagnosis and treatment.

The biogenesis of miRNAs has been extensively reviewed elsewhere [51] and is not the focus of this review. Briefly, miRNAs are generated endogenously through a series of steps, RNA polymerase II (or sometimes III) transcribes miRNAs in the nucleus as primary transcripts—pri-miRNA (~500–3000 nucleotides). Drosha (RNase) and DGCR8 (gene coding) shorten the pri-miRNA to ~70 nucleotides and build a stem-loop, which is called precursor miRNA (pre-miRNA). Exportin 5 transfers the pre-miRNA into the cytoplasm, where Dicer (RNase) cuts it into 22-nucleotide RNA duplexes. In most cases, the strand with less paired bases on the 5′ end is the mature miRNA, whereas the other strand is degraded. The mature miRNA builds a complex with the Argonaute 2 protein and the heterodimer of R2D2 & Dicer-2 proteins to form the RNA-induced silencing complex (RISC) [45,46,52,53]. The RISC complex is able to silence the expression of a target gene, by binding to the 3′ UTR of the target gene (mRNA). The binding inhibits the ribosome from translating the gene, which leads to reduced expression of the target gene [53,54].

There are three possible ways that miRNAs can negatively affect the translation of its target mRNA. If the base pairing between mRNA and miRNA is complete, it is most likely that degradation of the mRNA follows due to decreased steric hindrance. Secondly, by incomplete binding, the initiation site for the RNA-Polymerase is blocked leading to decreased mRNA transcription. The third option is that the miRNA-RISC complex translocates to so-called processing bodies (P-bodies), which lack ribosomal components and function as an mRNA storage and can mediate mRNA decay [55]. In contrast, some miRNAs are able to activate translation of their target mRNA when the cell is quiescent (not dividing and not preparing to divide), but this is the minority [56]. In these ways, miRNAs are able to knockout (or sometimes overexpress) genes that are important for the control of cellular homeostasis.

### 4.1. MicroRNAs in Cancer

In 2002 it was shown the first time that miRNAs are involved in cancer [57]. Calin *et al.* [57] discovered that miR-15a and miR-16-1 are located in a region that is frequently lost in leukaemia patients and that both miRNAs are deleted or significantly down-regulated in almost 70% of all chronic lymphocytic leukaemia patients. Since that initial discovery, cancer-associated miRNAs became classified as either oncogenic microRNAs (oncomiR) or tumour-suppressive microRNAs (tumour suppressor miR). These miRNAs are usually located in cancer-associated gene regions [57]. Whereas oncomiRs are frequently up-regulated in cancer, they target tumour suppressor genes for degradation and promote cancer cell growth; tumour suppressor miRs are usually down-regulated in cancer, they target oncogenes for degradation and have an anti-tumour function [58]. Inhibition of oncomiRs and overexpression of tumour suppressor miRs are therefore promising for targeted therapies in cancer. In almost all stages of the cancer process (cell cycle, apoptosis, invasion, angiogenesis), dysregulated miRNA expression has been found, when compared to normal tissue [59]. Altered miRNA expression profiles have been found in every type of human cancer (that has been studied so far) including colon cancer, brain tumours, lung cancer and breast cancer, where they mainly work as tumour suppressor miRs or oncomiRs [60,61]. These findings suggest that miRNAs may be possible biomarkers for early cancer detection [49,62,63].

### 4.2. miRNAs Involved in Triple Negative Breast Cancer

Over the last 10 years there have been multiple studies identifying miRNA changes associated with TNBC. Here we review and summarise the latest miRNA profiling (Table 1), functional (Table 2), and prognostic (Table 3) findings that have been implicated in the pathology of TNBC. We have also included studies in other breast cancer subtypes, where they analysed miRNAs targeting one of the three receptors (ER, PR, HER2, *i.e.*, miRs targeting these receptors may be responsible for the lack of receptor expression in TNBC) or those miRs associated with metastasis in other breast cancer subtypes, since these studies may allow us to gain further insight into miRNAs that may be involved in TNBC. While the majority of studies have compared TNBC against other breast cancer subtypes in an effort to determine miRNA signatures that can define this subtype, there have only been two studies which have focused on miRNA expression changes during tumour progression to identify biomarkers associated with the development of lymph node metastasis in TNBC samples. These studies performed miRNA profiling purely on TNBC samples (including our own [64]), identifying altered miRNA expression between tumour, matched normal and matched lymph node metastasis samples [64,65] and this represents an important area of investigation given the increased likelihood of TNBC to metastasize. A number of miRNAs have been identified and validated that target key genes involved in critical cellular functions. As an example, the miR-200 family targets *ZEB1*/*ZEB2*, *Suz 12*, *EphA2*, *MSN*, *FN1*, *TrkB*, *XIAP*, all of which are important for cell proliferation, invasion, and migration [66,67,68,69]. Multiple studies have revealed various miRNAs that specifically target the three missing receptors ER, PR, and HER2 as well as the breast cancer susceptibility gene *BRCA1* in TNBC development (see Table 1). The most recent study of prognostic miRNAs by Liu *et al.* (2015) identified a signature of four miRNAs that appeared to be associated with a good prognosis in TNBC (miR-374b-5p ↑, miR-218-5p ↑, miR-126-3p ↑, miR-27b-3p ↓). The following Tables summarise a broad overview of all three study types (profiling, prognostic and functional studies in cell lines) and their findings contributing to the current knowledge regarding miRNAs in TNBC. Overall, there is considerable inconsistency in the results of these studies and the effects of these miRNAs on various aspects of TNBC biology. This clearly suggests that there remains a need for better validation and reliability in the experimental conditions and subsequent analysis to define specific miRNAs as biomarkers of disease.

**Table 1:** MicroRNAs that have been associated with TNBC, the three receptors (ER, PR, HER2), and/or metastasis in profiling studies. MicroRNAs written in bold face have been analyzed in multiple studies. ↓ indicates down-regulation, ↑ indicates up-regulation.

miRNA	Result	Technology	References
miR-342, miR-299, miR-217, miR-190, miR-135b, miR-218	Associated with ER status	Expression profiling of 453 miRNAs, 29 breast cancer cases (mixed receptor status)	[70]
miR-520g, miR-377, miR-527-518a, miR-520f-520c	Associated with PR status		
miR-520d, miR-181c, miR-302c, miR-376b, miR-30e	Associated with HER2/neu status		
miR-532-5p, miR-500, miR362-5p, miR-502-3p	Located at Xp11.23 and present in TNBC, compared to other subtypes	miRCURY LNA arrays (2090 miRNAs analysed) 103 lymph node negative cases (mixed breast cancer subtypes)	[71]
Signature of 41 miRNAs	Associated with TNBC subtype		
Signature of 116 deregulated miRNAs	First study purely focused on miRNAs in TNBC	nanoString nCounter profiling (664 miRNAs analysed) 173 TNBC samples	[65]
miR-106b, miR-17/92 cluster, miR-200 family, miR-21, miR-155	Most up-regulated		
let-7b, let-7c, miR-126, miR-145, miR-205	Most down-regulated		
miR-424, miR-125a-5p, miR-627, miR-579, let-7g, miR-101	Associated with metastasis		
miR-130a	Second study purely on TNBC. Novel miRNAs, up-regulated in TNBC	Agilent miRNA microarrays (904 miRNAs analysed) 31 tumours, 13 lymph node metastasis, 23 normal adjacent tissues	[64,65]
miR-1280, miR-590-5p, miR-1308, miR-17*	Novel miRNAs, down-regulated in TNBC		
27 miRNA signature	Associated with lymph node metastasis, majority (25) are down-regulated		
miR-145, miR-205	↓ in TNBC (preferentially expressed in normal myoepithelial cells)	Tissue microarrays 100 TNBC samples	[72]
miR-17-92 cluster, miR-106b-25 cluster	Associated with oncogenic processes EMT, PI3K/Akt/mTOR, MYC, PTEN	miRNA and gene expression arrays, prediction software, data integration (miRNA arrays, (based on Sanger miRBase release 12.0, containing probes for 866 miRNAs) (29 mixed breast cancer subtypes))	[73]
miR-342, miR-299, miR-217, miR-190, miR-135b, miR-218	Markers for ER status	miRNA microarray, network algorithms, qPCR (453 miRNAs analysed) (mixed breast cancer subtypes)	[70]
miR-520g, miR-377, miR-527-518a, miR-520f-520c	Markers for PR status		
miR-520d, miR-181c, miR-302c, miR-376b, miR-30e	Markers for HER2		
miR-93	Associated with ER and PR status	miRNA profiling, qPCR (3 miRNAs analysed) (TaqMan MicroRNA Assays) (37 mixed breast cancer subtypes)	[74]
miR-200c, miR-205	Lower levels are associated with lymph node metastasis in TNBC	qPCR from tumour samples (16 miRNAs analysed) (32 TNBC samples)	[75]
miR-373, miR-10b	↑ regulated in cases with lymph node metastasis	qPCR from tumour samples (2 miRNAs analysed) (TaqMan MicroRNA Assays) (60 mixed breast cancer subtypes)	[76]

**Table 2:** MicroRNAs that have been associated with TNBC, the three receptors (ER, PR, HER2), and/or metastasis in functional studies. MicroRNAs written in bold face have been analyzed in multiple studies. ↓ indicates down-regulation, ↑ indicates up-regulation.

miRNA	Result	Functional Evidence	References
miR-200a/b	Tumour suppressor-miR/targets *ZEB1*/*ZEB2*, *Suz 12*, *EphA2*/plays role during differentiation in mammary epithelial cells	Cell culture experiment (differentiation) and qPCR (non-TNBC cell line HC11 mouse mammary)	[66]
miR-200c	Tumour suppressor-miR/targets *ZEB1*/*ZEB2*, *MSN*, *FN1*, *TrkB*/inhibits EMT and migration	Dual luciferase reporter assays, wound healing assays, cell-death ELISAs, and viability assays (non-TNBC cell lines: Hec50, AN3CA, MCF7; TNBC cell lines: MDA-MB-231, BT549)	[67,68]
miR-205	Tumour suppressor-miR/targets *E2F1*, *LAMC1*/supresses proliferation, cell cycle and tumour growth	Transfections, qPCR, colony formation assay, proliferation assay, cell cycle analysis, apoptosis assay, viability assay, senescence assay, western blot, chip assay (non-TNBC cell lines: HEK-293, MCF7, SAOS-2; TNBC cell lines: MDA-MB-231, BT549)	[77]
miR-203	Tumour suppressor-miR/targets *BIRC5*, *LASP1*/inhibits proliferation and migration	qPCR, transfection, proliferation and migration assays, luciferase reporter assay (non-TNBC cell lines: MCF-10A (normal); TNBC cell lines: MDA-MB-231, MDA-MB-468)	[78]
miR-31	Tumour suppressor-miR/targets *WAVE3*, *RhoA*, Radexin, *PRKCE*/suppresses metastatic potential, induction of apoptosis, increase of chemo-sensitivity	Transfection, qPCR, dual luciferase reporter assays, invasion assay, western blot, apoptosis assay, viability assay (non-TNBC cell lines: T-47D, MCF7, MCF-10A; TNBC cell lines: MDA-MB-231, MDA-MB-435, BT549)	[79,80]
miR-34a	Tumour suppressor-miR/targets *AXL*/inhibits migration	Target prediction, qPCR, dual luciferase reporter assays, DNA capture assay, western blot, proliferation and migration assays, cell cycle analysis (non-TNBC cell lines: MCF7, SK-BR-3, T47D; TNBC cell lines: MDA-MB-231, BT549, Hs578T)	[81]
miR-181a/b	Onco-miR/targets *Bim*, *ATM*/inhibits anoikisis, impairment of DNA double strand break repairs	Transfection, miRNA microarray, 3D cell culture, proliferation, migration and invasion assays, qPCR, dual luciferase reporter assays, tumour growth and metastasis assay, cell cycle analysis (non-TNBC cell lines: NMuMG, MCF7, HEK 293GP, SUM159PT, OVCAR,HT29, PANC1, SK-Br-3; TNBC cell lines: MDA-MB-231, MDA-MB-468)	[82,83]
miR-146	Onco-miR/targets *BRCA1*/effects *BRCA1*-mediated proliferation and homologous recombination	Target prediction, transfections, qPCR, northern blot, western blot, dual luciferase reporter assays, proliferation assay (TNBC cell lines: MDA-MB-436, MDA-MB-157)	[84]
miR-182	Onco-miR/targets *PFN1*/increases proliferation and invasion, decreases apoptosis	Transfections, proliferation assay (MTT and flow cytometry), apoptosis assay, invasion assay, dual luciferase reporter assays, western blot (TNBC cell line MDA-MB-231)	[85]
miR-200 family	Inhibits cancer cell migration, invasion, if low → poor response to chemotherapy and radiotherapy	qPCR, dual luciferase reporter assays, immunoblot and immunofluorescence assay, migration assay, ChIP assay, viability assay, clonogenic assay, western blot (non-TNBC cell lines: NMuMG, HeLa, MCF7; TNBC cell lines: MDA-MB-231)	[86,87,88,89]
Let-7 family	Tumour suppressor-miRs ↓ in TNBC/target onco-genes *RAS*, *MYC*, *HMGA2*	c. elegans, mice, cell culture, transfections	[90,91]
miR-15a,b, miR-16, miR-128	Target *Smurf2* (tumour suppressive ubiquitin) which down-regulates retinoblastoma (tumour suppressor) in TNBC	Immunohistochemistry, qPCR, transfection (non-TNBC cell lines: MCF-10A (normal breast), MCF9, T47D, SK-Br-3, BT747; TNBC cell lines: MDA-MB-231, MDA-MB-468, BT549, MDA-MB-436, DU4475)	[92]
miR-200c	Targets X-linked inhibitor of apoptosis (*XIAP*), what then suppresses proliferation in TNBC	Transfections, qPCR, colony formation assay, proliferation assay, flow cytometry, western blot, luciferase reporter assays, mice tumour model (non-TNBC cell lines: MCF-10A (normal breast); TNBC cell lines: MDA-MB-231)	[69]
miR-221	Onco-miR/promotes tumourigenesis in TNBC/if knocked-down cell cycle progression and induction of apoptosis is inhibited	Transfections, qPCR, immunoblotting, proliferation, migration, invasion, and apoptosis assays, cell cycle analysis, mice tumour analysis (non-TNBC cell lines: SKBR3, MDA-MB-361, T47D, ZR75-1, MCF-7; TNBC cell lines: MDA-MB-231, Hs-578T, BT-20, and MDA-MB-468)	[93]
miR-31	Antimetastatic-miR/when ↓ regulated in TNBC more metastases/down-regulated due to promoter methylation	qPCR, bisulfite-modified DNA for methylation analysis, DNA sequencing, methylation specific PCR (non-TNBC cell lines: MCF-10A (normal breast), MCF7, SKBR3, T47D; TNBC cell lines: MDA-MB-231, BT549, MDA-MB-4355)	[94]
miR-200b	Targets protein kinase Cα and suppresses metastasis in TNBC	qPCR, transfection, luciferase reporter assays, migration assay, mouse xenograft model, immunohistochemistry, western blot, pulldown assay, MTT assay, colony formation assay (non-TNBC cell lines: MCF-7, T-47D, BT-474, SKBR-3; TNBC cell lines: MDA-MB-468, BT-20, Hs578T and BT-549, MDA-MB-453)	[95]
miR-22, miR-27a, miR-206, miR-221/222, miR-302c	Associated with ER signalling and endocrine resistance	Immunohistochemistry, qPCR, transfections, clonogenicity assay, microarray, western blot, viability assay, luciferase reporter assays (non-TNBC cell lines: MCF-7, BT-474, T47D, SK-BR-3; TNBC cell lines: MDA-MB-231)	[96,97,98,99]
miR-125b, miR-134, miR-193a-5p, miR-199b-5p, miR-331-3p, miR-342-5p, miR-744*	Associated with HER2 signalling and trastuzumab resistance	luciferase reporter assays, northern and western blot, proliferation, migration and invasion assays, microarray, qPCR, transfections (non-TNBC cell lines: MCF-10A (normal breast), SK-BR-3, KPL-4, JIMT-1, MCF-7, BT-474; TNBC cell lines: MDA-MB-231)	[100,101,102]
miR-23b/27b/24 cluster	Promotes metastasis by targeting prosaposin (=metastasis-suppressive gene)	Microarrays, qPCR, migration assay, tumour xenografts, luciferase reporter assays, western blot (non-TNBC cell lines: HeLa; TNBC cell lines: MDA-MB-231, 67NR, 168FARN, 4TO7, 66cl4, 4T1)	[103]

**Table 3:** MicroRNAs that have been associated with TNBC, the three receptors (ER, PR, HER2), and/or metastasis in prognostic studies. MicroRNAs written in bold face have been analyzed in multiple studies.

miRNA	Result	Predictive/Prognostic	References
miR-200 family	Inhibits cancer cell migration, invasion, if low → poor response to chemotherapy and radiotherapy (non-TNBC based studies & TNBC study [88])	predictive	[86,87,88,89]
miR-21	Onco-miR/associated with poor prognosis/↑ expressed in TNBC	prognostic	[104]
miR-155	Onco-miR/associated with poor prognosis, angiogenesis, tumour growth, metastases/controlled epigenetically by *BRCA1*↑ expressed in TNBC	prognostic	[105]
miR-16, miR-155, miR-374	Prognostic miR/if ↑ associated with better prognosis (overall survival) (TNBC based study)	prognostic	[65]
miR-125b	Prognostic miR/if ↓ associated with poor prognosis (overall survival) (TNBC based study)		
miR-125b, miR-655, miR-421	Risk associated miRs/associated with distant disease free survival (TNBC based study)		
miR-16, miR-374a,b, miR-497	Protective miRs/associated with distant disease free survival (TNBC based study)		
miR-210	↑ regulated in TNBC compared to ER+ breast cancers/associated with early relapse/low levels are associated with better disease free survival in TNBC	prognostic	[104,106,107]
miR-34b	Associated with p53-pathway/negative correlation with disease free survival and overall survival (TNBC based study)	prognostic	[108,109]
miR-376b, miR-409-5p, miR-410miR-193a-3p	Associated with worse breast cancer specific survival (TNBC based study)	prognostic	[73]
miR-16-2* ↑, miR-381 ↓, miR-409-5p ↓, miR-766 ↑	Associated with better distant metastasis free survival (TNBC based study)		
miR-766, miR-33b*, miR-550, miR-1539, miR-548d-5p, miR-16-2*, miR-563, miR-155*	Positively correlation with prognosis (TNBC based study)		
miR-193a-3p, miR-432, miR-376b, miR-381, miR-409-5p, miR-410	Negatively correlated with prognosis (TNBC based study)		
miR-342, miR-150	miRNAs for good prognosis (TNBC based study)	prognostic	[110]
miR-27b, miR-210, miR-144	miRNAs for poor prognosis (TNBC based study)		
miR-21	Onco-miR/↑ regulated in TNBC/associated with poor prognosis, shorter recurrence-free survival and increased proliferation	prognostic	[111,112]
miR-155	Onco-miR ↑ regulated in TNBC/targets tumour suppressor *VHL* and promotes angiogenesis/associated with poor prognosis	prognostic	[113]
miR-200b-3p ↑, miR-190a ↑, miR-512-5p ↓	In this combination associated with better response to chemotherapy (TNBC based study)	predictive	[114]
miR-155-5p, miR-21-3p, miR-181a-5p, miR-181b-5p, miR-183-5p	↑ regulated in TNBC/associated with chemoresistance	predictive	[115]
miR-10b-5p, miR-451a, miR-125b-5p, miR-31-5p, miR-195-5p, miR-130a-3p	↓ regulated in TNBC/associated with chemoresistance	predictive	[115]
miR-155, miR-30e, miR-27a, miR-493	Biomarkers dividing TNBC into low and high level risk groups	prognostic	[116]
miR-10b	↑ in TNBC/promotes tumour invasion and metastasis/shorter progression free and overall survival/by targeting *HoxD10* (which depresses expression of prometastatic gene *RhoC*)	prognostic	[76,117,118,119]
miR-374b-5p, miR-218-5p, miR-126-3p	When ↑ in TNBC associated with good prognosis	prognostic	[120]
miR-27b-3p	When ↓ in TNBC associated with good prognosis	prognostic	

### 4.3. MicroRNAs and Metastasis/Epithelial to Mesenchymal Transition (EMT)

As discussed previously, TNBCs have an increased propensity to metastasize and the majority of deaths from this disease are a result of distant disease. Epithelial-mesenchymal-transition (EMT) has become the focus of research into the metastatic process. EMT describes a process by which epithelial cells lose their adhesive qualities resulting in increased mobility. The process of EMT was first described as a feature of embryogenesis [121]. There are three types of EMT. Type one describes the process during implantation, embryogenesis and organ development. Type two is involved in wound healing, to generate fibroblasts after tissue injuries. The third type of EMT describes the relationship between EMT and cancer progression [122].

**Figure 1:**
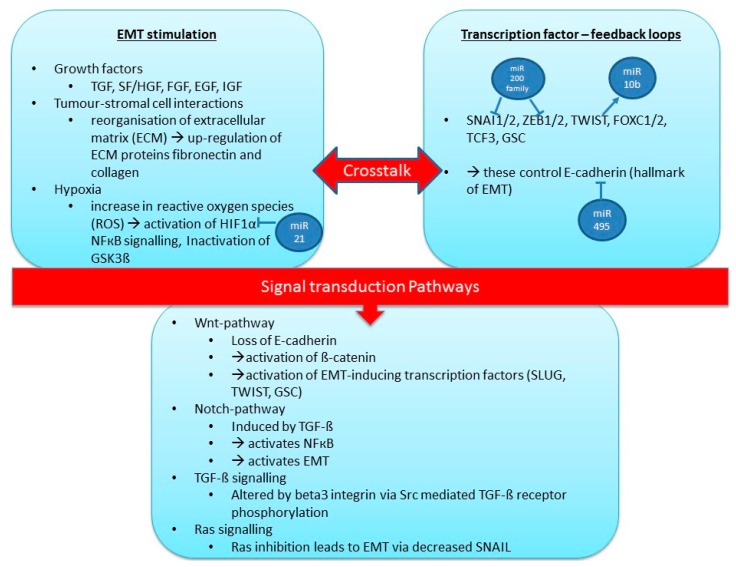
Overview of the process of epithelial-mesenchymal transition (EMT).

EMT induction involves multiple genes/pathways, for example the genes Src [123], Ras [124], Ets [125], integrins [126], Wnt/β-catenin [127,128], Notch [129] and others [122] have been associated with this process. A summary of these pathways is shown in Figure 1 and has been graphically illustrated in a review by Kalluri and Weinberg in [122]. Briefly, EMT can be initiated by growth factors, tumour-stromal cell interaction, or hypoxia. These stimuli create feedback loops with transcription factors, which for the most part, control E-cadherin (a key molecule in EMT). All of these pathways involve multiple genes, all of which could be affected by epigenetic change, such as miRNA expression changes, thereby altering critical pathways involved in this process. A summary of the key miRNAs and their regulation of EMT-related target genes in breast cancer development is shown in Table 4.

There are multiple reports revealing how EMT is affected by various miRNAs [130,131,132,133,134,135,136]. All of them demonstrate the inter-connectivity between miRNAs, EMT and cancer progression. In particular, the miR-200 family has roles in proliferation, migration and invasion [130,132,135,136] and has been well-studied in breast cancer research. Basal-like breast tumours are known to have more mesenchymal and EMT features than other subtypes supporting their more aggressive nature [137]. Basal like breast tumours have lower levels of the miR-200 family than luminal or HER2 over-expressing subtypes [138], this also causes higher levels of their target genes (*SNAI1*/*2*, and *ZEB2*). Of the miR-200 family, miR-200c shows the strongest association with the histopathology and disease course, this miRNA is also almost non-existent in TNBC [65,139]. Various studies have also shown that the miR-200 family has dual functions in breast cancer progression [140,141]. These studies reveal in primary breast tumours that miR-200 levels are low, which would lead to increased *ZEB1*/*2* expression and E-cadherin down-regulation [142] and are associated with EMT initiation and subsequent invasion into the blood stream. At the secondary site, miR-200 levels rise and initiate mesenchymal-epithelial transition (MET) culminating in metastatic colonisation.

In TNBC the miR-200 family has also been shown to sensitise cell lines to cell death and to inhibit metastatic growth through inhibition of protein kinase (PKCα) [95,143]. Other members of the miRNA-200 family, miR-221/222 have the opposite effect compared to miR-200c, where low miR-221/222 expression improves the differentiation status [135]. miR-130b works in a similar fashion, if it’s expression is high, the level of EMT is low [133]. However, there are many more miRNAs that play a role in metastasis that have yet to be fully characterised.

An overview of miRNAs that are known to be involved in metastatic spread and EMT as well as their targets and the target gene expression is provided in Table 4. This table does not contain all genes and microRNAs known to be involved in metastatic spread and EMT but it does show that this is a complex process with multiple influencing factors.

More recently there has been some controversy regarding the dependency of metastasis on EMT. There are an increasing amount of studies that have identified mechanisms by which cancer cells migrate and metastasise that are EMT-independent [144,145,146,147]. During EMT-independent metastasis, neoangiodenic vessels infiltrate the tumour and build a connection to capillary endothelia of distant organs, where metastatic growth occurs [144]. These EMT-independent mechanisms are not well understood and are not the focus of this review. Nevertheless, a number of genes are involved in both processes, including *SNAIL*, *TWIST*, *ZEB1/2* [144,145,146,147].

**Table 4:** Overview of microRNAs that are known to be involved in metastatic spread and epithelial-mesenchymal transition (EMT) in breast cancer.

EMT-Regulation	miRNA	Comments	References
Pro-EMT	miR-21	Associated with invasive and metastatic breast cancer; regulates EMT and *HIF-1a*	[148,149,150]
	miR-29	Activates EMT by down-regulating peroxidasin homologue (cell adhesion molecule); down-regulation of DNA-methylation of tumour-suppressor genes; increasing chemosensitivity; targets EMT regulator *N-myc*	[151,152]
	miR-10b	Targets *Tiam1*-mediated Rac activation, which controls cell-cell adhesion and EMT through E-cadherin, leads to increased cell invasion and migration; Is activated by transcription factor *TWIST* (binds to promoter of miR-10b); expression increases during *TGF*-β induced EMT	[118,153,154]
	miR-9	Is up-regulated in breast cancer, represses cadherin-1, which regulates cell adhesion and proliferation	[155,156]
	miR-206	Suppresses proliferation, targets *ER*, *SRC-1*, *SRC-2*, *GATA-3* (all estrogen signalling molecules)	[157,158,159]
	miR-221/222	Increases proliferation in ER positive cell lines, targets *ER*, *p27*, *p57*	[97,160,161]
	miR-495	Targets E-cadherin, *JAM-A*, and *REDD1*	[162,163]
	miR-181	Targets *PHLAD1* and *ATM*, associated with reduced survival in TNBC	[83,164]
Pro-EMT (sometimes anti-EMT)	miR-17/92 cluster	Can act as tumour suppressor and oncogene, depending on microenvironment, mostly pro-metastatic, targets ER and *SRC-3*	[165,166]
Anti-EMT	miR-130a	Targets ER, *c-MET* (onco-gene), down-regulates miR-221/222	[167]
	miR-145	Acts as tumour suppressor, targets ER and *MUC-1* (supports cell invasion)	[168,169,170]
	miR-7	Targets *SETDB1* → reduction of *STAT1*, *Myc*, *Twist*, and miR-9	[171]
	miR-375	Targets *SHOX2* and *IGFR*, which leads to suppression of EMT	[172]

### 4.4. Circulating miRNAs in Breast Cancer

There is an urgent need for less invasive diagnostic and prognostic biomarkers for breast cancer and in particular for TNBC. The majority of biomarkers to date have been developed from tumours comparing gene/miRNA expression against healthy control samples [65], or different tumour subtypes [30,173]. More recently circulating miRNAs have become a focus for the development of less invasive biomarkers. Circulating miRNAs can be cell-free single miRNAs, transported in exosomes or other microvesicles and are present in blood, plasma, serum, urine or other body fluids [174]. Recently, there has been an increase in studies focusing on circulating miRNAs in breast cancer, which have been summarised in Table 5. miR-155 has been the most widely studied circulating miRNA in breast cancer. It is upregulated in the serum of breast cancer patients compared to healthy controls [175,176,177]. miR-155 expression has been associated with ER/PR/HER2 expression [177]. The expression levels of miR-155 can also discriminate between primary breast cancer and metastatic breast cancer, as the expression decreases significantly in metastatic breast cancers compared to primary cancer and healthy tissue [178]. As a result of these findings, it has been classed as a stable biomarker for breast cancer, confirmed through a meta-analysis of circulating miRNAs in breast cancer [179]. Further to this, it has been found to be down-regulated in breast cancer patients after surgical tumour removal [176]. Very recently *TRF-1* has been identified as a target gene of miR-155 [180]. *TRF-1* has a sheltering function for chromosome telomeres, which has decreased expression as a result of high miR-155 expression leading to a decrease in genomic stability, metastasis-free survival and relapse-free survival in ER-positive breast cancer patients. Inhibition of miR-155 results in an improvement of telomere function and an increase in genomic stability [180]. Circulating miR-155 is now considered a key regulator in breast cancer development and progression. Nevertheless, there are many other circulating miRNAs with important functions in breast cancer, which have been summarised in Table 5. Similar to other miRNA studies, there is a lack of consistency between studies. A stricter definition of tumour suppressor and oncogenic miRs is required so that significant miRNAs in body fluids and tissue samples can be made to fully appreciate the biological role these nucleic acids have in tumour development. These findings must be replicated in different cohorts before we can call these miRNAs biomarkers. Nevertheless, the majority of these miRNAs have also been identified in tumour samples, which supports their relevance in the involvement in the tumour development and progression (Table 1, Table 2 and Table 3).

**Table 5:** Overview of circulating microRNAs identified in breast cancer patients. ↓ indicates down-regulation, ↑ indicates up-regulation.

MicroRNA	Study Findings	References
miR-34a, miR-93, miR-373, miR-21, miR-155, miR-155, miR-181b, miR-24, miR-19a, miR-21, miR-106, miR-155, miR-29a, miR-21, miR-20a, miR-21	Are up-regulated in breast cancer compared to healthy controls	[175,176,177,178,179,181,182,183]
miR-299-5p, miR-411, miR-126, miR-199a, miR-335, miR-181a, miR-1304	Are down-regulated in breast cancer compared to healthy controls	[177,184,185]
miR-17, miR-155 (↑ in primary), miR-10b, miR-210, miR-214, miR-18b, miR-103, miR-107, miR-652, miR-101, miR-372, miR-373	Discriminating primary tumour from metastatic tumour	[178,182,186,187,188,189]
miR-373, miR-17, miR-34a, miR-21, miR-126, miR-155, miR-199a, miR-335	Associated with ER/PR/HER2 status	[177,178]
miR-210, miR-214, miR-10b, miR-373	Associated with lymph node metastasis; miR-214 targets *PTEN* (tumour suppressor)	[76,182,187]
miR-200b, miR-18b, miR-103, miR-107, miR-652, miR-155	Associated with survival	[180,188,190]
miR-210 ↓ (surgery), miR-214 ↓ (surgery), miR-155, miR-181b, miR-24 ↓ (surgery), miR-19a ↓ (therapy)	Levels of miRNA-expression change after surgery/therapy	[176,182,187]
miR-141, miR-200a,b,c, miR-203, miR-210, miR-375, miR-810 ↑, miR-768-3p ↓	Altered in patients with circulating tumour cells (CTC) compared to patients without CTC	[190]
miR-210	Higher in patients with residual disease than patients who achieved pathological complete response; Correlates with sensitivity to trastuzumab	[187]
miR-16, miR-21, miR-199a-5p	Lower in TNBC compared to non-TNBC; miR-199a-5p associated with tumour stage in TNBC	[191]
miR-373	Exosomal levels higher in TNBC compared to luminal breast cancer	[186]
miR-127, miR-197, miR-222, miR-223	Target *CXCL12*; are transposed via gap junctions from bone marrow to breast cancer cells and also through exosomes; → leading to cell quiescence, might contribute to dormancy of bone marrow metastasis	[192]
miR-223	Macrophages secret microvesicles that contain this miRNA, promoting cell invasion	[193]
miR-222	Chemoresistance is transmitted between breast cancer cells via exosomes with specific miRNAs	[194]
miR-105	Is secreted in exosomes from metastatic breast cancer cells; targets *ZO-1* (tight junction protein) → destroying tight junctions (barrier for metastasis). It’s over-expression induces metastasis	[195]
miR-155	Targets *TRF-1* (telomere sheltering function); high levels are associated with low *TRF-1*, metastasis-free survival, and relapse-free survival in ER+ cases. Reducing miR-155 improves telomere function and genomic stability	[180]

### 4.5. miRNAs as Therapeutics

The above mentioned studies clearly show the importance and involvement of miRNAs in cancer initiation, development, and progression and also in chemotherapy resistance. As such, they have become a focus in drug development studies for targeted therapies. Many studies have shown the tumour suppressor or oncogenic functions of miRNAs through knock-downs or overexpression experiments in cell lines and more recently in mice (see Table 2). One of the challenges is the delivery of these miRNAs to the affected tissue, without affecting the healthy tissue. There are multiple challenges for the delivery of miRNAs including biological barriers, toxicity of the miRNA, tissue specificity and the monitoring of the delivery. All of these have been reviewed in [196]. There are different mechanisms for miRNA delivery; earlier methods were based on intravenous injections of anti-miRNA oligonucleotides into mice [197]. Nevertheless, these “naked” oligonucleotides were not stable and were easily degraded by endogenous RNAs. The addition of cholesterol conjugated 2′-O-methyl groups to these oligonucleotides increases their stability and produces what is commonly referred to as an antagomiR. Krutzfeldt *et al.* were the first to demonstrate the effectiveness of antagomiRs in silencing their target miRNA (miR-16, miR-122, miR-192 and miR-194) in liver, lung, kidney, heart, intestine, fat, skin, bone marrow, muscle, ovaries and adrenals [198]. An antagomir to miR-10b was also shown to inhibit metastatic growth in a mouse mammary tumour model [117]. Further development led to so-called “locked nucleic acid” (LNA) oligomers [199]. These have an increased miRNA binding affinity, greater stability and reduced toxicity due to a ribose moiety that is locked into a C3′-endo conformation via an addition of a methylene bridge [199]. Recently, a study by Xing *et al.* showed LNA targeting BCAR4 (long-noncoding RNA) strongly suppresses breast cancer metastasis in a mouse model [200]. Another approach is miRNA sponges, which are vector-encoded molecules [201]. The advantage of these sponges is that they contain multiple binding sites, which enables them to inhibit multiple miRNAs simultaneously. Furthermore, they can be stably integrated in the genome and to create stable cell lines or transgenic animal models. Nevertheless, the vector size and poor distribution in the body makes them almost unusable in humans, which explains why there are no breast cancer trials that have used miRNA sponges.

The above methods have shown the progression of developing potential therapeutics which can inhibit oncogenic miRNAs, but there are also several approaches for increasing the levels of tumour-suppressive miRNA. Adeno-associated viruses (AVV) are known to have a high transferring ability, low immunorejections and long-term gene expression [202]. miR-26a is a tumour suppressor miR that is known to be down-regulated in multiple cancers, including breast cancer. It has been shown that over-expression of miR-26a via AVV suppresses tumorigenesis with no signs of hepatoxicity or dysregulation of endogenous miRNAs (murine liver cancer model) [203]. A very recent study by Trepel *et al.* focused on the systemic delivery of dual-targeted AAV vectors for the treatment of multifocal breast cancer to overcome collateral tropism, which is frequent with the use of AAV vectors [204]. By including miRNA-regulated transgene cassettes they achieved stronger, completely tumour-specific transgene expression. Another method to increase miRNA levels is via nanoparticles, which are small positively charged structures that can be used to transport negatively charged miRNAs to the target tissue [205]. The major advantages of these nanoparticles are that they improve miRNA stability and release the miRNAs slowly for prolonged mRNA targeting, which also helps to avoid possible immunogenicity (associated with AAVs) [206]. Further development of these nanoparticles has made it possible to design multifunctional RNA nanoparticles, as shown in the recent study by Shu *et al.* [207]. They developed a multifunctional RNA nanoparticle that delivered anti-miR-21, as well as an EGFR targeting aptamer and a fluorescent imaging module (Alexa647) into a TNBC mouse model. The particle bound strongly to the tumour and showed little or no accumulation in healthy tissue eight hours after injection and significantly repressed tumour growth at a low dose. These studies show how research is developing to bring the use of miRNA-based therapies in clinics a realisation. Until this time, further validations and the first breast cancer clinical trials are necessary to enhance the targeting function and decrease possible side effects.

## 5. DNA Methylation

The basis of epigenetic change is centred around the modification of CpG islands, histones and nucleosome positioning. Many cellular processes are influenced by epigenetic change, including gene expression, cellular differentiation, genomic imprinting and embryogenesis [208]. Epigenetic differences can be observed even in genetically identical twins who can suffer from diverse genetically driven diseases, such as cancer, as a result of different DNA methylation profiles [209,210]. Feinberg and Vogelstein were the first to report on epigenetic change in cancer as they found colorectal cancer cells were hypo-methylated compared to normal tissue [211]. DNA hypo-methylation leads to oncogene activation and chromosome instability culminating in tumour development. Conversely, hyper-methylation has been shown to inhibit tumour suppressor genes, thereby releasing cells from their normal physiological control.

DNA methylation is associated with the addition of a methyl group to a cytosine base in DNA and is usually associated with genomic stability, but is also associated with the control of gene expression via an alteration in the transcriptional accessing of transcriptional start sites. Embryonic stem cells use this biochemical process to differentiate into tissue specific cells, which is usually irreversible [212].

DNA methylation mostly occurs on so called CpG islands, which are DNA regions with at least 200 bases that consist of at least 50% C + G content [213]. The majority of human promoters are associated with CpG islands and are usually unmethylated, only a few become methylated during development or cell differentiation. The DNA methyltransferase (DNMT) converts the cytosine bases into 5-methylcytosine, which generally leads to gene silencing. The different kinds of DNMTs are also necessary to maintain DNA methylation after cells traverse the cell cycle. DNMT1 will copy the methylation pattern and replicate it to the daughter DNA strand and is therefore called a maintenance enzyme [214]. In mammals the enzymes DNMT3a and DNMT3b are responsible for the initial DNA methylation [215]. There are multiple mechanisms in which DNA methylation can inhibit gene expression. It can lead to binding of methyl-CpG-binding domain proteins (MBD), which then recruit histone modifying and/or chromatin remodelling complexes to the methylated site that inhibit gene expression, by forming a more compact and inactive chromatin [216]. DNA methylation can also inhibit the binding of transcription factors to the promoter; nevertheless a recent study suggests that this does not occur frequently [217,218]. Recently, it has been shown that not only CpG islands, but also CpG shores (regions close to CpG islands with less C + G content) can be methylated, which may be a form of tissue specific methylation and gene expression inhibition [219].

Many gene expression-related diseases, including breast cancer, are affected by DNA methylation. The right level of methylation is especially important during early development to secure key processes like X-chromosome inactivation and genomic imprinting as well as the development of tissue specific cells from embryonic stem cells. Indeed incorrect DNA methylation levels during development can result in disease or death [214,220].

### 5.1. Epigenetic Gene Inactivation during Breast Cancer Development and Progression

Early studies generally focused on known cancer related genes (mostly tumour suppressor genes) as their methylation would lead to gene silencing. An example for this is a study by Berman *et al.* who identified that *p16* is methylated and therefore inactivated in early breast cancer [221]. Another example is the epigenetic inactivation of *SLIT3* in 12 out of 29 (41%) breast cancer cell lines and 5 out of 32 (16%) primary tumours [222]. The finding that their promoter methylation results in blocking transcription supports the notion that they are required to inhibit cellular proliferation. Yan *et al.* took the first global approach of DNA methylation analysis in breast cancer by using CpG island arrays, where 28 breast cancer samples were compared to 28 normal samples to assess their global hypermethylation status and determine if this was associated with tumour grade [223]. They confirmed that 9% of the tested 1104 CpG sites showed increased methylation in tumour samples compared to the normal samples. DNA methylation has also been used as a marker for breast cancer hormone receptor (HR) expression. The methylation level of *ESR1* (gene for ERα) can be used as a predictor for the PR status and the methylation of *PGR* (gene for PR) can predict ER status [224]. Genome-wide methylation studies have now been performed by: (1) methylated DNA immunoprecipitation (MDIP) followed by hybridisation to high density oligonucleotide arrays [225], or next generation sequencing [226]; (2) Next-generation genome-wide sequencing of bisulfite-converted DNA [227] and (3) Illumina bead chip arrays (27 K or 450 K array format) [228].

Multiple studies have aimed at identifying biomarkers for early breast cancer by comparing ductal carcinoma *in situ* (DCIS) with invasive ductal carcinoma (IDC). This has been reviewed by Pang *et al.* [229]. Muggerud identified *ABCB1*, *FOXC1*, *GSTP1*, *MGMT*, *MCH1*, *PPP2R2B*, *PTEN* and *RASSF1A* as potential biomarkers for early breast cancer detection in DCIS as these genes already show altered methylation in DCIS compared to normal breast tissue, at the same level as in IDCs [230]. Since the review there have been further studies. The methylation of *MINT17*, *MINT31*, *RAR*ß*2*, and *RASSF1A* has been found to increase throughout disease development from normal breast to ductal hyperplasia, to atypical ductal hyperplasia, to DCIS, to IDC [231]. A genome-wide study comparing DCIS and IDC to normal identified 5000 differentially methylated genes comparing normal to DCIS and 1000 genes comparing DCIS to IDC [232]. These markers may provide potential prognostic value for future breast cancer patients.

### 5.2. DNA Methylation for Breast Cancer Subtype Classification

DNA methylation can also be used to improve current breast cancer classification. There is a distinct difference between methylation and gene expression profiles of breast cancer such that not all methylation profiles fit within the same molecular subtype. Nevertheless, this might lead to an improvement of the current classification, which could also improve future treatment options and breast cancer diagnosis/prognosis. Multiple studies used a panel of 807 cancer-related genes to classify breast cancer patients into subtypes by analysing their DNA methylation [233,234,235]. All three studies support that there is a difference in the epigenetic profile of breast cancer compared to their respective gene expression pattern (defining the molecular subtype). Bediaga *et al.* identified subtype specific methylation profiles for basal-like, luminal A, and HER2-overexpressing breast cancers [233]. A similar study by Holm divided 189 breast cancer samples into luminal A, luminal B, and basal-like with the methylation profile of 807 cancer-related genes. HER2-enriched and normal-like breast cancers were distributed between them [234]. A third study by Ronneberg clustered 80 breast cancer tumours into three groups using the methylation profile of the 807 cancer-related genes. These groups were distinct for ER status, TP53 status, HER2 status and tumour grade [235]. One of the first genome-wide studies to determine whether DNA methylation could be used to classify breast cancer samples into intrinsic subtypes used the MDIP assay on 33 breast cancer samples, to compare genome-wide methylation with the expression of 25,500 transcripts. Again, it confirmed that the epigenetic profile does not necessarily group the samples into the same breast cancer subtypes as gene expression profiling [236]. This study identified an association of the DNA methylation profile with the *BRCA* status of breast cancer samples. More recently Conway *et al.* (analysis of 935 CpG sites in 517 breast tumours from the Carolina Breast Cancer Study) identified breast cancer DNA methylation to be associated with hormone receptor status, subtype and *TP53* mutation status [237]. It was found that *BCR*, *C4B*, *DAB2IP*, *MEST*, *RARA*, *SEPT5*, *TFF1*, *THY1* and *SERPINA5* were all hypermethylated in hormone receptor negative, basal-like, and/or *TP53* mutated tumours, whereas *FABP3*, *FGF2*, *FZD9*, *GAS7*, *HDAC9*, *HOXA11*, *MME*, *PAX6*, *POMC*, *PTGS2*, *RASSF1*, *RBP1* and *SCGB3A1* were hypermethylated in hormone receptor positive, luminal A and/or p53 wild-type tumours. Earlier this year Stefansson *et al.* [238] performed Infinium 450K arrays on 212 tumours and discovered that luminal B breast cancer show CpG island promoter methylation, whereas basal-like tumours show hypomethylation events in gene bodies. Therefore, they named the two epigenetic breast cancer subtypes Epi-LumB and Epi-Basal, which are also associated with unfavourable clinical parameters and reduced survival [238].

As discussed earlier, there is an urgent need for biomarkers that can be measured by a less invasive technique than from tumour specimens. Circulating miRNAs provide one option but nevertheless, DNA methylation profiles from patient serum may provide an alternative. Even though DNA methylation is cell type specific it is possible to extract circulating tumour cells from serum and perform DNA methylation analysis on them. This has been done by Jing *et al*. They were able to show that the CIMP (CpG island methylator phenotype) is different from patient serum compared to healthy controls [239]. A seven gene hypermethylation profile was identified by Radpour *et al.* by comparing triple matched samples from tumour tissue, normal tissue and serum. These seven genes were *APC*, *BIN1*, *BMP6*, *BRCA1*, *CST6*, *P16* and *TIMP3*, which were hypermethylated in tumour tissue and serum compared to normal tissue [240]. Nevertheless, further studies are needed to validate these markers and to assess their robustness.

### 5.3. DNA Methylation in TNBC

There have been few studies focusing on DNA methylation in TNBC, and until earlier this year, there were no whole genome DNA methylation analyses. Stirzaker *et al.* identified a DNA methylation signature for TNBC patients that divided patients into one of three groups based on their disease outcome (poor, medium, and good) using *The Cancer Genome Atlas* data (TCGA) [241]. They identified that TNBC patients with low levels of tumour DNA methylation in the gene signature had the best prognosis, followed by high levels of methylation with an intermediate prognosis and lastly, patients whose tumours had medium methylation levels had the worst prognosis. Further to this, they identified a gene methylation signature that separated TNBC from non-TNBC cases [241]. A number of studies have analysed the methylation status of *BRCA1*, a key player in breast cancer and TNBC. One of the first studies to identify that the *BRCA1* promoter was methylated in TNBC came from Veeck *et al.* [242] in 2010. They suggested that this was a marker for the effectiveness of PARP inhibitors. They showed that the sensitivity of TNBC breast cancer cell lines to PARP inhibitors was increased when *BRCA1* was methylated [242]. *BRCA1* promoter methylation has also been associated with low expression of pRb and high expression of p76 [243]. A study by Watanabe *et al.* showed that methylation of the homologous recombination DNA repair genes *BRCA1* and *RNF8* was significantly higher in TNBCs than luminal breast cancers. At the same time, *BRCA1* methylation was higher in patients with pathological complete response than in non-responders to neoadjuvant chemotherapy. The opposite effect was seen for *RNF8* [244]. The *BRCA1* gene plays an important role in breast cancer. TNBC cases have similar histopathological and molecular features as those breast cancers that result from germline *BRCA1* mutations. However, only 10%–20% of TNBCs have a *BRCA1* mutation, which led Sharma *et al.* to hypothesise that these similarities are due to epigenetic inactivation of *BRCA1* [245]. Within their cohort of 39 TNBC patients, they identified that 30% of them had *BRCA1* promoter methylation, which led to silencing of this gene. Further, they showed that the overall survival for patients with *BRCA1* promoter methylation was only 36%, whereas for patients without *BRCA1* promotor methylation, the overall survival was 77% [245]. In contrast to this, Xu *et al.* showed that TNBC patients with *BRCA1* methylation are more sensitive to adjuvant chemotherapy and that this is associated with better survival when compared to TNBC patients without *BRCA1* methylation [246]. Their study showed an increased 10-year disease free survival of 78% in patients with *BRCA1* methylation, compared to 55% in patients without *BRCA1* methylation. The 10-year disease specific survival also increased in patients with *BRCA1* methylation (85%) compared to patients without *BRCA1* methylation (69%). This was confirmed by Ignatov *et al.* [247], where they also showed that *BRCA1* methylation is only associated with disease-free survival in TNBC but not in non-TNBC cases (*p* = 0.009 compared to *p* = 0.322).

## 6. Interactions between miRNAs and Epigenetic Mechanisms

It has been shown that epigenetic mechanisms of gene inactivation can be controlled by other epigenetic processes. miRNAs have been shown to target DNMT enzymes and influence the DNA methylation process [248,249]. On the other hand, important proteins for the biogenesis of miRNAs can be methylated and decrease the number of transcribed miRNAs.

The majority of miRNAs have been found to be located within the intronic regions of protein-coding genes [250], which are so-called host genes, and as such they can be co-regulated [251]. Nevertheless, miRNAs also have their own promoters, which can be near/within CpG islands within the same intron where the miRNA is located. A study by Wee *et al.* identified that approximately 60% of 93 breast cancer associated miRNAs are within 5 kb of a CpG island [252]. This suggests that miRNAs can be transcribed from their own promoter, and that this promoter can be regulated by DNA methylation. This has been shown in multiple cancer studies [253,254,255,256]. As an example, Lehmann *et al.* have shown that miR-9-1, miR-124a3, miR-148, miR-152 and miR-663 are epigenetically inactivated through hyper-methylation in breast cancer [257]. Additionally, miR-31 has been shown to be methylated in TNBC and this leads to an increase in the expression of its pro-metastatic target genes (*RhoA* and *WAVE3*) [94].

As mentioned above, miRNAs can also control the epigenetic machinery. Fabbri *et al.* were the first to identify that the miR-29 family directly targets the DNA methyltransferases DNMT3a and DNMT3b [249]. The miR-29 family is downregulated in lung cancer and DNMT3a and3b are up-regulated, this can be reversed by miR-29 over-expression. This also causes re-expression of methylation-silenced tumour suppressor genes (*FHIT*, *WWOX*). Later these miRNAs were referred to as epi-miRs [257]. The miR-148 family can also target DNMT3b, resulting in decreased DNA methylation levels and altered splicing of DNMT3b [248]. Interestingly, as mentioned above, miR-148a is also epigenetically regulated through promoter hyper-methylation [253], suggesting an epigenetic feedback loop. In summary, there are a number of studies focusing on the interaction between epigenetic mechanisms-miRNAs and methylation. A better understanding of these interactions will improve the knowledge of cancer development and progression, which will lead to improved diagnostic and prognostic markers.

## 7. Conclusions

In conclusion, the majority of biomarkers for TNBC have been identified through gene and microRNA expression analysis. There is still a lack of concordance between these studies and limited understanding of the influence that DNA methylation plays in the regulation of TNBC development and progression. There is a need for studies to correlate the findings between miRNA/gene expression profiles and epigenetic profiles of TNBC, in order to develop a better understanding of the disease process and identify robust biomarkers.
